# Single-cell transcriptomic analysis reveals the immunosuppressive status of NK cell subpopulations in TNBC

**DOI:** 10.1371/journal.pone.0354524

**Published:** 2026-07-23

**Authors:** Yang Liu, Wen-Ming Cao, Ying Jin, Weizhen Xu

**Affiliations:** 1 Department of Clinical Trial, Zhejiang Cancer Hospital, Hangzhou, Zhejiang, China; 2 Hangzhou Institute of Medicine (HIM), Chinese Academy of Sciences, Hangzhou, Zhejiang, China; 3 Department of Breast Medical Oncology, Zhejiang Cancer Hospital, Hangzhou, Zhejiang, China; Weill Cornell University, UNITED STATES OF AMERICA

## Abstract

Triple-negative breast cancer (TNBC), lacking expression of the estrogen receptor (ER), progesterone receptor (PR), and human epidermal growth factor receptor 2 (HER2), has clinical features that include high invasiveness, limited response to current immunotherapies, heightened risk of recurrence, and poorer overall prognosis. Central to these adverse clinical outcomes is the tumor microenvironment (TME), where the presence of immature natural killer (NK) cells with lower cytotoxic capacity has been associated with disease progression. Through a comprehensive analysis of publicly available single-cell transcriptomic data from breast cancer, we identified three NK cell subtypes within TNBC and non-TNBC tissues, named NK_XCL1, NK_FCGR3A, and NK_ISG15. Compared to non-TNBC, the presence and cytotoxic activity of NK_FCGR3A cells in TNBC were markedly diminished, primarily due to a substantial increase in the expression of inhibitory receptors like NKG2A (KLRC1). NK_ISG15 cells in TNBC show higher expression of type I interferon signaling genes. Through cell-type cross-talk analysis, we found that the feature of NK_FCGR3A cells in TNBC was mediated by myeloid cells with the HLA-E-KLRC1/HLA-E-CD94:NKG2A signal pathway. Notably, through integrating with public cancer transcriptomic data, we found higher expression of ISG15 is associated with poor prognosis, while higher expression of NK_FCGR3A signature genes is correlated with favorable prognosis. Collectively, these findings and the markers identified offer valuable insights into the mechanisms of immune evasion in TNBC, underscoring potential targets for developing therapeutic strategies that may improve patient outcomes.

## Introduction

Breast cancer is one of the leading malignancies affecting women worldwide. With approximately 2.3 million new cases in 2020, it ranks as the most common cancer among women globally [1,2]. Triple-negative breast cancer (TNBC), which constitutes roughly 20% of all breast cancer cases [3], is particularly challenging. TNBC is defined by the absence of the estrogen receptor (ER), progesterone receptor (PR), and human epidermal growth factor receptor 2 (HER2). Patients with TNBC encounter a higher risk of recurrence, estimated at 10–15% within the first 1–2 years post-treatment [4,5], and face a significantly reduced survival time after metastasis, often only 10–13 months [3]. The absence of ER, PR, and HER2 markers in TNBC limits the effectiveness of targeted therapies, which are readily available for non-TNBC subtypes. Although there have been advancements in T cell-based immunotherapies in conjunction with chemotherapy, they fail to benefit approximately 30–40% of patients with early-stage TNBC [6,7]. This underscores the critical need for research into the molecular mechanisms and pathways driving TNBC, with the goal of developing more effective precision therapies for this aggressive form of cancer.

Given the limited efficacy of T cell-based immunotherapies in a subset of patients with TNBC, NK cells have emerged as important cytotoxic effectors and potential therapeutic targets. Recent single-cell studies have reported that breast-cancer-infiltrating NK cells can be broadly categorized into two major transcriptional and functional populations: 1) cytotoxic NK cells, also referred to as NK_FGFBP2, NKT, or NK_cyto_, which highly express cytotoxic genes such as GZMB and NKG7 and can mediate antibody-dependent cellular cytotoxicity (ADCC) against target cells; and 2) chemokine-secreting or regulatory NK cells, also referred to as NK_XCL1 or NKrest, which secrete chemotactic factors that promote dendritic cell (DC) recruitment and may support downstream T-cell cross-priming and antigen-specific immune responses [8–10]. The compromised cytotoxic function of NK cells has been implicated in the progression of breast cancer [11]. This diminished activity is often linked to decreased levels of activating receptors like NKG2D and FCGR3A, as well as increased expression of the inhibitory receptor NKG2A [12, 13]. The abundance and activation status of tumor-infiltrating NK cells have also been associated with treatment response in breast cancer. For example, FCGR3A-expressing NK cells may contribute to the efficacy of antibody-based therapies such as trastuzumab through antibody-dependent cellular cytotoxicity, whereas NK-cell activation or dysfunction may influence the response to immune checkpoint blockade [12,13]. Nonetheless, the expression patterns of genes commonly found in NK cells have been associated with the survival outcomes of breast cancer patients [14–16]. In particular, the identification and roles of distinct NK cell subsets present within TNBC tumors, and how they may relate to the progression of TNBC, remain incompletely defined up to this point.

Studies indicate that TNBC exhibits notable distinctions from non-TNBC. For instance, TNBC is characterized by the lowest proportion of NKT:FCGR3A cells among breast cancer subtypes [9]. Additionally, the more aggressive progression and poorer prognosis of TNBC, compared to non-TNBC, might be mainly due to large numbers of lower cytotoxic capacity NK cells [11,17]. These findings have led us to pursue an in-depth analysis of NK cell diversity and dysfunction in the TNBC microenvironment. In this study, we analyzed publicly available approximately 460,000 single-cell transcriptomes from breast cancer to characterize NK cells within the TNBC tumor microenvironment. We identified three NK cell subtypes within TNBC and non-TNBC tissues, named NK_XCL1, NK_FCGR3A, and NK_ISG15 according to their representative marker genes. Our results indicate a statistically significant decrease in the proportion of NK_FCGR3A cells in TNBC, as well as enhanced inhibitory ligand-receptor interactions with DCs and macrophage cells. Importantly, NK_ISG15 cells are significantly enriched with genes related to type I interferon signaling. These interferons, in turn, promote the overexpression of HLA-E, which strengthens the receptor-ligand interaction between NKG2A (KLRC1) and HLA-E, ultimately suppressing the anti-tumor activity of NK cells. Additionally, we observed a negative correlation between ISG15 gene expression level and patient outcomes, whereas a positive correlation was found between NK_FCGR3A cell gene signatures and survival in breast cancer patients. This comprehensive analysis enables us to better predict outcomes for TNBC patients and to pinpoint potential new targets for therapy.

## Methods

### Data integration

Single-cell RNA-seq data (10x Genomics) was processed using the Seurat R package (R 4.1.2, Seurat 4.1.1) [18]. Low-abundance genes were removed by filtering out any gene detected in ≤3 cells. Cells with fewer than 200 or more than 6,000 detected genes, or with >15% of reads mapping to mitochondrial genes, were excluded from downstream analysis. The filtered expression data were then normalized using Seurat’s NormalizeData function to reduce technical variability while preserving biological signal. To integrate data from multiple samples and correct for batch effects, we employed Seurat’s Canonical Correlation Analysis algorithm. In this integration workflow, each sample’s dataset was first normalized independently using the SCTransform method, and the 2,000 most variable genes from each sample were selected as features for integration. Normalized integration local inverse Simpson’s index (norm-iLISI) was calculated by LISI R package (R 4.1.2, LISI 1.0)

### Data clustering, differential expression analysis and annotation

All dimensionality reduction, clustering, and differential expression analyses were carried out with Seurat. Principal component analysis (PCA) was performed on the integrated dataset using Seurat’s RunPCA function to capture the major sources of variation. For clustering, a shared nearest neighbor (SNN) graph was constructed by finding each cell’s 15 nearest neighbors in PCA space (FindNeighbors on the top 15 PCs). Cell clusters were then identified by applying the Louvain community detection algorithm [19] via Seurat’s FindClusters function (resolution = 0.4). Finally, we used the RunUMAP function in Seurat to generate a two-dimensional embedding for visualization.

To identify cluster-specific markers and other differentially expressed genes (DEGs), we utilized Seurat’s FindAllMarkers function with default parameters. Genes were considered significant markers if they showed a minimum log2 fold-change of 0.5 and were detected in at least 25% of cells in the relevant cluster; only positive markers were reported. Each cell cluster was annotated to a specific cell type by examining the expression of well-known marker genes.

### Gene set enrichment analysis

We performed Gene Set Enrichment Analysis (GSEA) to identify pathways and processes enriched in the differential expression results. The GSEA software from the Broad Institute (http://software.broadinstitute.org/gsea/index.jsp) was used to evaluate whether the DEGs in NK cells between the TNBC and non-TNBC groups were significantly associated with curated gene sets by default parameters.

### Gene Ontology enrichment analysis

For each cell type (and condition group), we compiled the list of DEGs and carried out Gene Ontology (GO) enrichment analysis to interpret their functional significance. Enriched GO terms for each set of DEGs were identified using the clusterProfiler R package (v4.2.2) [20] with default settings. The org.Hs.eg.db annotation database was applied to map gene identifiers to GO terms. Results of the GO enrichment analysis were visualized as bar plots, illustrating the top significantly enriched GO categories for each cell population or comparison.

### Ligand-receptor interaction analysis

Intercellular communication between cell populations was examined using CellPhoneDB (v2.0.0) [21]. This algorithm infers potential ligand–receptor interactions by statistically evaluating the co-expression of ligand genes in one cell type and their corresponding receptor genes in another cell type. We applied CellPhoneDB to our single-cell expression data to reveal significant cell–cell interactions and to compare interaction patterns between the TNBC and non-TNBC groups.

### Pseudotime trajectory analysis

To explore developmental trajectories and lineage relationships among cells, we performed pseudotime analysis using two independent R packages, Slingshot [22] and Monocle [23]. Monocle was applied to the NK cell subset, using the NK_XCL1 subpopulation as the designated root or starting point for trajectory inference. In parallel, Slingshot was used to infer lineage pathways within the NK cells. Both methods were run with default parameters.

### Gene regulatory network analysis

We employed the SCENIC pipeline (v1.3.1) [24] to investigate transcriptional regulatory networks and regulon activity in NK cell subtypes under TNBC and non-TNBC conditions. To ensure comparability, we randomly down-sampled each NK cell subtype to a maximum of 2,000 cells per condition, mitigating biases from unequal cell numbers. We also filtered the gene set for SCENIC to include only genes that were differentially expressed between TNBC and non-TNBC and detected in at least 100 cells, focusing the analysis on relevant regulators.

For each condition, SCENIC was run following its standard three-step workflow. First, co-expression networks were inferred using the GENIE3 algorithm to predict candidate transcription factor (TF)–target relationships from the expression data. Next, RcisTarget was used to perform cis-regulatory motif enrichment, refining the predicted regulons by identifying which TFs have enriched binding motifs in the putative target genes’ promoters. Finally, AUCell was applied to quantify regulon activity scores for each cell, indicating the activity level of each TF’s target gene set (regulon) in every single cell. We then binarized the regulon activity matrix using the runSCENIC_4_aucell_binarize function.

To identify key regulators that distinguish TNBC from non-TNBC conditions, we compared regulon activity between the two groups. For each NK cell subtype, the top 10 differentially active regulons (transcription factors) were determined using the Wilcoxon rank-sum test. We considered a regulon to be significantly different between conditions if it exhibited an average log2 fold-change > 0.25, p-value < 0.01, and was active in >25% of cells in at least one condition.

### Statistical analysis

All statistical analyses were performed using appropriate tests in R. For comparisons of gene expression between two groups of cells (e.g., DEGs between conditions), we used the Wilcoxon rank-sum test. Differences in cell-type proportions between conditions were evaluated with a one-way ANOVA. In cases of multiple hypothesis testing, such as GO term or KEGG pathway enrichment, p-values were adjusted using the Benjamini-Hochberg procedure to control the false discovery rate.

### Ethics statement

Informed consent was not required for this study because datasets presented in this study can be found in public databases.

## Results

### Integration of single-cell transcriptome in tumor tissues from breast cancer patients

**Fig 1:**
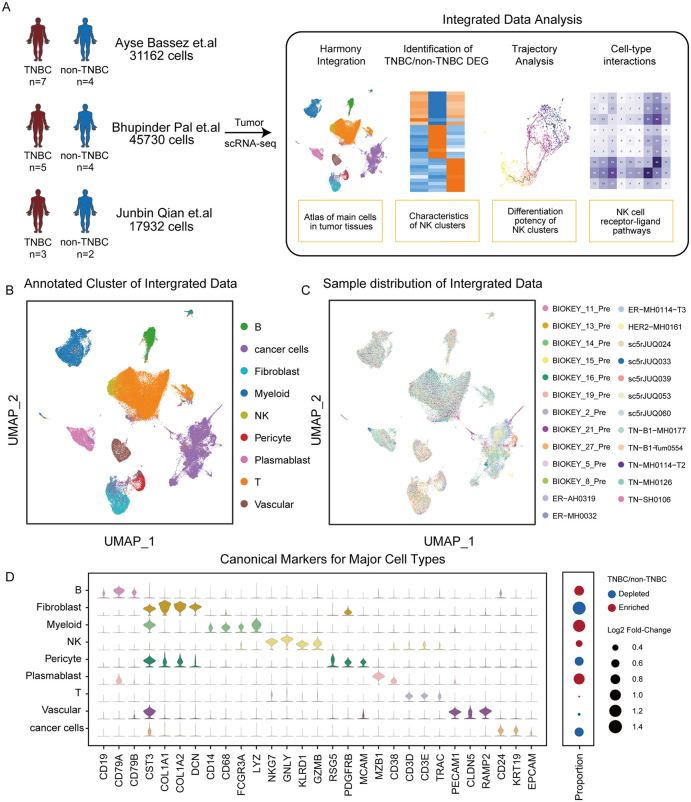
Single cell transcriptome data integration to identify TME in breast cancer. (A) A schematic outline depicting the workflow for data collection from published literature and subsequent integrated analysis. Numbers indicate the number of samples of different studies and the number of single-cell transcriptomes analyzed. (B) Uniform manifold approximation and projection (UMAP) visualization of cell types in breast cancer tumor tissues from 25 samples. Cells are colored by cell types. (C) UMAP visualization of individual samples illustrates no obvious batch effect in this integrated atlas. (D) Violin plots of canonical markers (columns) for cell types (rows). Dot plots of the proportion shifts of cell types in TNBC, compared to non-TNBC. The circle color indicates enrichment (red) or depletion (blue), and the circle size indicates the values of log2 (TNBC/non-TNBC).

To investigate the heterogeneity of single-cell transcriptome profiles in breast cancer tumor tissues, we integrated single-cell RNA-seq data from three public studies involving 130 samples (S1A Fig). Specifically, data from Ayse Bassez et al. [10] include 38 TNBC samples, 8 HER2^+^ samples, and 38 ER samples. Data from Bhupinder Pal et al. [25] include 8 TNBC samples, 6 HER2^+^ samples, and 18 ER^+^ samples. Data from Junbin Qian et al. [8] include 8 TNBC samples, 3 HER2^+^ samples, 1 Luminal B-like sample, 1 Luminal A-like sample, 1 Luminal HER2^+^ sample. After removing cell doublets using Scrublet and filtering out low-quality cells, approximately 460,000 single-cell transcriptomes were retained for downstream analysis (S1B Fig). We then integrated the filtered single-cell transcriptomes using Seurat’s Canonical Correlation Analysis to mitigate batch effects (S1C Fig). To further quantify the effectiveness of integration, we calculated the normalized integration local inverse Simpson’s index (norm-iLISI) using study origin as the batch variable, which is recommended by a recent study [26]. The norm-iLISI increased from 0.10 before integration to 0.40 after integration, supporting improved cross-study mixing after CCA correction, although residual study-specific variation may not have been completely eliminated. 27 distinct cell clusters were identified through normalization and clustering analysis, displayed using uniform manifold approximation and projection (UMAP) (S2A Fig). We identified NK cell-enriched clusters using canonical markers (*e.g.*, NCAM1, NKG7, GNLY, KLRF1, KLRD1, KLRK1, GZMH, NCR1, IL2RB, PRF1, GZMA, TRDC) (S2B-C Fig). After excluding samples with insufficient NK cell representation (less than 1% of all cells or less than 30 cells), treatment effects, and outliers, we refined our dataset to include 25 untreated patient samples—15 with TNBC samples and 10 with non-TNBC samples (3 HER2^+^, 6 ER^+^, 1 Luminal B-like) (Fig 1A).

A subsequent round of integration and cluster analysis classified B cells, cancer cells, fibroblast cells, myeloid cells, NK cells (totaling 1997), pericyte cells, plasmablast cells, T cells, and vascular cells (a total of 94824 cells in nine cell types) (Fig 1B). UMAP visualization of individual samples illustrates no obvious batch effect (Fig 1C), thus validating the cell-type identifications against traditional markers [8,10,25] (Fig 1D). In our integrated dataset, the proportions of myeloid cells and NK cells were increased, while the proportion of cancer cells was decreased in TNBC relative to non-TNBC. Concurrently, we observed a significantly higher infiltration of B cells in the TNBC group, a finding that is highly consistent with previous single-cell transcriptomic profiling reports [27] (Fig 1D). This approach underpinned a robust single-cell transcriptomic landscape and ensured the precision of cell type identification in the tumor tissues studied.

### Functional profiling of NK cell subpopulations in breast tumors

**Fig 2:**
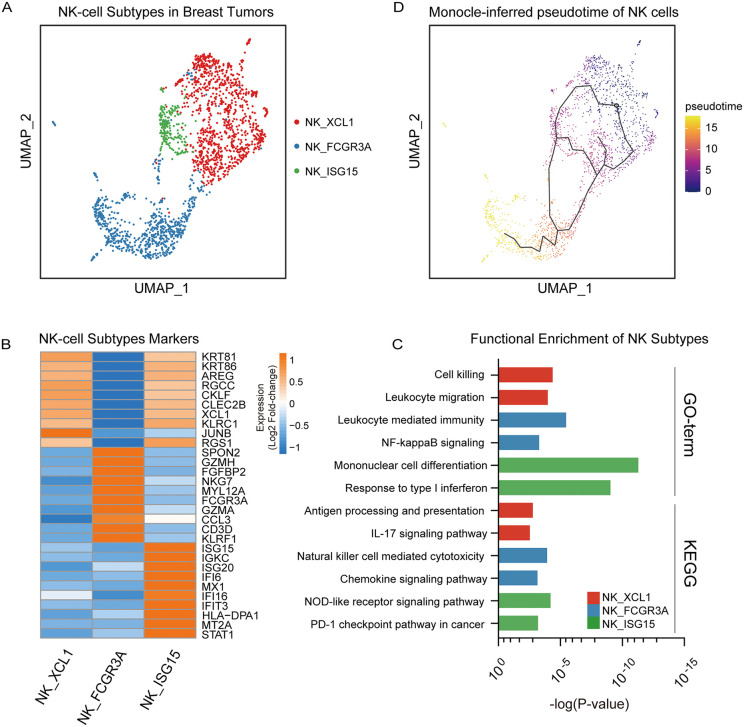
Functional profiling of NK cell subpopulations in breast tumors. (A) UMAP visualization of NK subtypes in tumor tissues. Different NK subtypes are color-coded. (B) Heatmap of the gene expression of the differentially expressed genes (DEGs) (top 10) for each NK subtype. (C) GO (top) and KEGG (bottom) pathway annotation of gene signatures of NK_XCL1 cells, NK_FCGR3A cells and NK_ISG15 cells. (D) Development trajectory of NK subtypes is depicted in the pseudo-temporal trajectory diagram.

In our in-depth analysis of NK cells from 15 TNBC and 10 non-TNBC breast tumor samples, we identified three transcriptionally distinct populations, designated NK_XCL1 (XCL1-high chemokine-associated NK-cell state), NK_FCGR3A (FCGR3A/CD16-high cytotoxic NK-cell state), and NK_ISG15 (ISG15-high interferon-stimulated NK-cell state) (Fig 2A). Differential expression analyses revealed 287 subtype-specific genes across these subsets (Fig 2B, S1 Table). We defined NK cell subpopulations using marker genes meeting all criteria: 1) log2 (Fold Change) > 0.5 (vs. other NK clusters); 2) Adjusted p-value < 0.05 (Benjamini-Hochberg correction); 3) Expressed in at least 25% of cells within the target subpopulation. NK_XCL1 cells were characterized by elevated expression of XCL1, CLEC2B, and KLRC1, consistent with chemotactic and antigen-presentation-associated programs. NK_FCGR3A cells demonstrated high expression levels of cytotoxic markers, such as GZMH, FGFBP2, and FCGR3A, underscoring their potent cytotoxic capabilities (Fig 2C). The function of NK_XCL1 cells and NK_FCGR3A cells has been identified by previous studies. However, NK_ISG15 cells were linked with type I interferon signaling and NOD-like receptor pathways, indicating their involvement in innate immune responses [28] (Fig 2C).

To explore transcriptional relationships among NK cell subsets, we performed pseudotime analysis using Monocle. The inferred continuum suggested a trajectory-like arrangement from NK_XCL1 toward NK_FCGR3A cells (Fig 2D), which is broadly consistent with maturation-associated transcriptional programs reported in previous studies [29–31]. However, pseudotime analysis reflects transcriptional similarity rather than experimentally validated lineage relationships, and therefore should be interpreted as indicating putative state transitions rather than direct evidence of developmental lineage. Slingshot analysis yielded a broadly similar topology (S3A Fig). Collectively, these results support the presence of three transcriptionally distinct NK-cell states in breast cancer, with NK_ISG15 showing a heightened interferon-associated inflammatory program and NK_FCGR3A cells displaying pronounced cytotoxic features.

**Fig 3:**
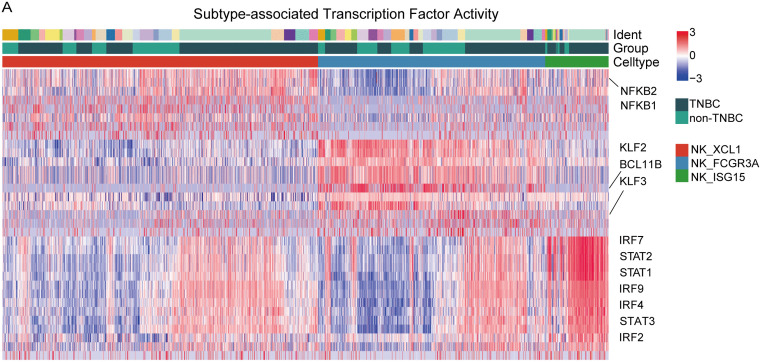
Transcription factors profiling of NK cell subpopulations in breast tumors. (A) Heatmap to show the expression regulation by transcription factors of NK subtypes in breast cancer, as estimated by pySCENIC, highlighting subtype-associated regulatory programs.

We also explored transcription factors (TFs) in NK cells that may regulate NK cells’ function gene. Utilizing the SCENIC algorithm, we mapped the TF landscape across these subtypes, uncovering a shared functional annotation. Our analysis revealed an upregulation of NF-κB in NK_XCL1, which may be associated with the regulation of perforin (PRF1) expression in NK cells. BCL11B and KLFs—reported as crucial regulators in NK cell maturation—were upregulated in NK_FCGR3A. In contrast, STATs and IRFs were predominant in NK_ISG15 cells, linking them to a strong interferon gene signature (Fig 3A). Collectively, these results support the presence of three transcriptionally distinct NK-cell states in breast cancer, with NK_ISG15 showing a heightened interferon-associated inflammatory program and NK_FCGR3A cells displaying pronounced cytotoxic features.

### Heterogeneity of NK cells in TME of TNBC and non-TNBC

**Fig 4:**
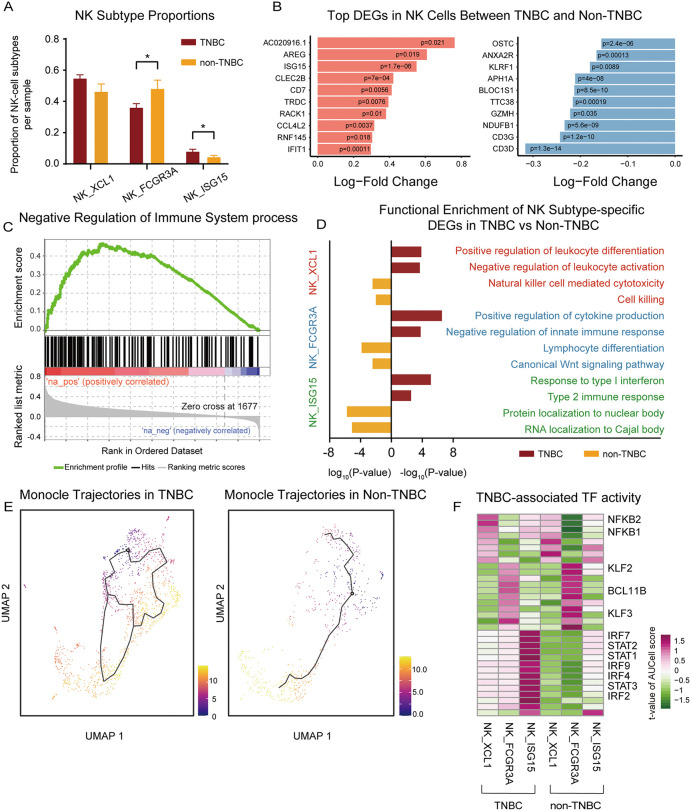
Heterogeneity in the molecular features of NK cells in TME of TNBC and non-TNBC. (A) Boxplots of the cell proportions of each NK subtype in TNBC and non-TNBC. (B) Boxplots show the fold-change and p-value of top DEGs’ gene expression between TNBC and non-TNBC NK cells. (C) GSEA shows highly statistical enriched in negative regulation of immune system process in TNBC, compared with non-TNBC. Genes are ranked by their enrichment scores. (D) GO and KEGG pathway annotation of gene signatures of NK_XCL1 cell, NK_FCGR3A cell, and NK_ISG15 cell in TNBC and non-TNBC. (E) Development trajectories of NK subtypes in TNBC (left) and non-TNBC (right) are depicted in the pseudo temporal trajectory diagram. (F) Heatmap to show the t-value for the area under the curve score of expression regulation by transcription factors of NK subtypes in TNBC (left half) and non-TNBC (right half), as estimated by pySCENIC, highlighting condition-associated differences in NK-cell regulatory programs.

Previous studies have demonstrated that among breast cancer subtypes, TNBC stands out with the lowest proportion of NK_FCGR3A cells [9]. Additionally, the proportion of NK_XCL1 cells in TNBC surpasses that of NK_FCGR3A cells [9] and increased numbers of NK_XCL1 cells in TNBC tumors are correlated to poor overall survival [11,17]. In our study, similar trends in the disparity of cell percentages between NK_XCL1 cells and NK_FCGR3A cells were detected for TNBC samples. Compared to non-TNBC, the TNBC group exhibited a marked decrease in the proportion of cytotoxic NK_FCGR3A cells (P = 0.035), accompanied by a marginal increase in less cytotoxic NK_XCL1 and NK_ISG15 cells (Fig 4A). Furthermore, through differential expression and GO/KEGG enrichment analysis, we found that NK cell DEGs were notably enriched in pathways associated with C-type lectin receptor signaling, and the MAPK pathway, such as CLEC2B and TRDC. (Fig 4B, S4A Fig). Importantly, gene set enrichment analysis (GSEA) [32] also reveals that the immune system was significantly negatively regulated in TNBC NK-cells compared to non-TNBC NK-cells (Fig 4C). We further examined the differences among three subgroups (S2 Table). The primary function of NK_XCL1 cells from the TNBC group is positive regulation of leukocyte differentiation and negative regulation of leukocyte activation. NK_FCGR3A cells from the TNBC group exhibited significant upregulation of KLRC1 and KLRD1 genes [9,33], encoding the C-type lectin inhibitory receptors NKG2A and CD94, respectively, which suppress cytotoxic function [17,34] (Fig 4D). Additionally, NK_ISG15 cells in TNBC were enriched in type I interferon response genes, including those for interferon β and γ, and the TNF signaling pathway (Fig 4D). This enrichment suggests a heightened inflammatory response in TNBC compared to non-TNBC [11,35], potentially influencing disease progression through the MAPK pathway [36].

Our comparative pseudotime analysis of NK cells in TNBC and non-TNBC suggests different organizations of NK-cell transcriptional states between the two groups. Although both groups display an inferred continuum from NK_XCL1 to NK_FCGR3A cells (Fig 4E, S3B-C Fig), monocle identifies a TNBC-associated branch involving NK_ISG15 cells. Because pseudotime analysis captures transcriptional similarity rather than experimentally validated lineage relationships, the observed branching pattern does not constitute direct evidence of developmental divergence. Instead, NK_ISG15 may represent a distinct IFN-responsive functional state that emerges under the inflammatory conditions of TNBC, rather than a bona fide intermediate developmental stage. Together, these pseudotime analyses indicate an altered organization of NK-cell transcriptional states in TNBC, contributing to the reduced presence of cytotoxic NK_FCGR3A cells in TNBC.

Lastly, transcription factor analysis within the TNBC cohort highlighted a pronounced upregulation of IRFs and STATs in NK_ISG15 cells, critical for driving interferon gene expression, coupled with a downregulation of BCL11B and KLFs (maturation-associated transcription factors) in NK_FCGR3A cells (Fig 4F). These findings correlate with the decreased presence and impaired function of mature NK cells in TNBC, alongside an increase in cytokine-secreting NK cells, contributing to the distinctive immunological profiles observed between TNBC and non-TNBC cases.

### Differential receptor-ligand dynamics of NK cells in TNBC *vs.* non-TNBC

**Fig 5:**
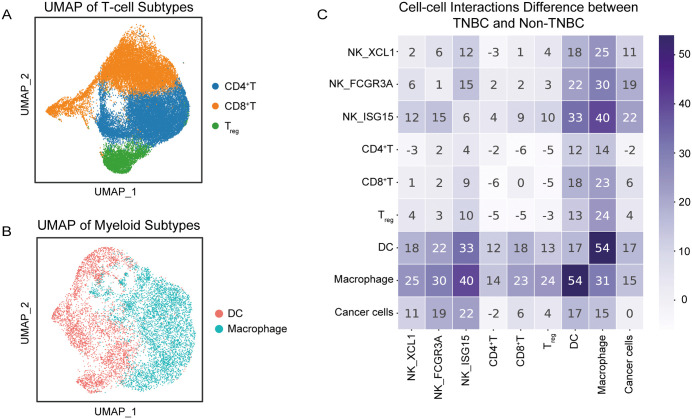
Heterogeneity of NK cells in receptor-ligand dynamics between TNBC and non-TNBC. (A) UMAP visualization of T subtypes in breast cancer. Different T subtypes are color-coded. (B) UMAP visualization of myeloid subtypes in breast cancer. Different myeloid subtypes are color-coded. (C) Difference in the number of significant CellPhoneDB interactions comparing TNBC and non-TNBC.

Pan-cancer analyses have revealed intricate interactions among T cells, myeloid cells, and NK cells [37], we next examined receptor-ligand communication patterns. Firstly, we re-clustered the T cells and myeloid cells. Subclustering of T cells and myeloid cells, guided by known markers and differentially expressed genes (S5C Fig), resulted in the identification of CD8^+^T, CD4^+^T, and T_reg_ clusters, and macrophage and DC myeloid clusters (Fig 5A-B and S5A-B Fig). Significant variations in the infiltration levels of T_reg_ cells within tumor tissues between TNBC and non-TNBC groups are found (P = 0.02) (S5D Fig). Macrophage infiltration proportions showed no significant difference between TNBC and non-TNBC, nor did dendritic cell infiltration proportions (S5E Fig). To discern the specific differences in these interactions within TNBC and non-TNBC cohorts, we utilized CellPhoneDB to construct a cell-cell communication network. In breast cancer, NK cells exhibited robust interactions with macrophages and DCs (S6A-B Fig), aligning with previous research findings [10,37]. Notably, these interactions were significantly more pronounced in the TNBC group compared to non-TNBC (Fig 5C).

**Fig 6:**
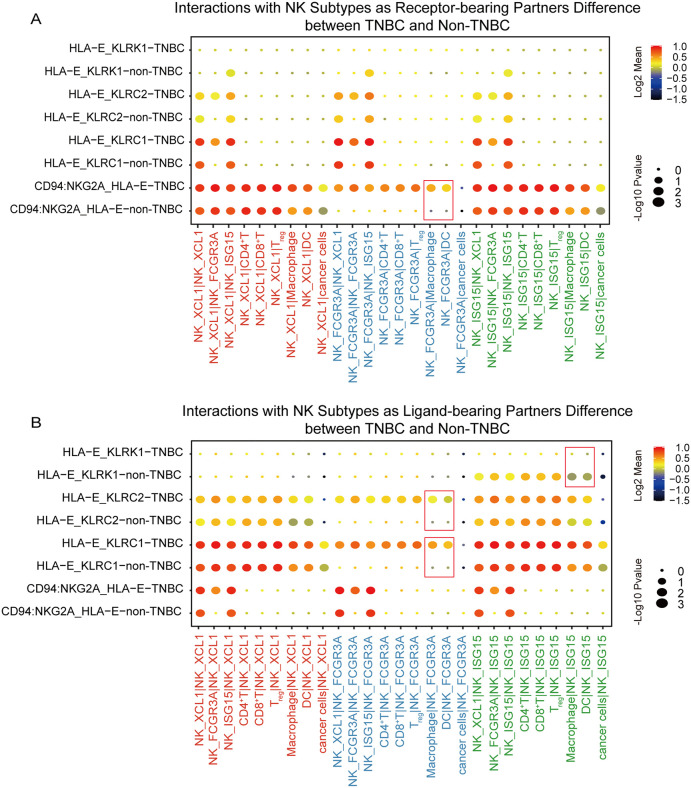
Directional analysis of NK-centered HLA-E/NKG2-family ligand–receptor interactions. (A-B) Dot plots showing condition-specific significance and interaction strength of selected NK-centered ligand–receptor interactions inferred by CellPhoneDB in TNBC and non-TNBC. Interactions were examined between NK subtypes and T-cell subtypes (CD8 + T cells, CD4 + T cells, and Treg cells), myeloid subtypes (DCs and macrophages), and cancer cells. (A) Interactions in which NK subtypes were positioned as receptor-expressing partners. (B) Interactions in which NK subtypes were positioned as ligand-expressing partners. Dot size represents −log10(P value), and dot color represents log2 mean expression of the ligand–receptor pair.

Our investigation revealed substantial variability in classical NK cell receptor-ligand pathways such as HLA-E-KLRC1, HLA-E-CD94:NKG2A, and HLA-E-KLRK1 (NKG2D), and others (Fig 6A-B). In the TNBC group, NK_FCGR3A cells showed elevated expression of the receptors KLRC1 and KLRD1 (CD94), recognizing HLA-E on DCs and macrophages. The formation of HLA-E-KLRC1 and HLA-E-CD94:NKG2A complexes, with HLA-E observed at significantly higher levels in macrophages in TNBC (P = 6.87E-13), suggests a mechanism for the inhibition of NK_FCGR3A cell cytotoxicity in TNBC. Despite a rise in the activation pathway HLA-E-KLRC2 (NKG2C) within the TNBC group, the low affinity of NKG2C for HLA-E relative to NKG2A [38]—and the non-significant difference in KLRC2 expression between TNBC and non-TNBC groups (t test P = 0.899)—implies a continuation of reduced cytotoxic activity in TNBC-associated NK_FCGR3A cells. Functionally, this dense interaction network suggests that macrophages and DCs act as primary immunosuppressive hubs in the TNBC microenvironment. The pronounced HLA-E-KLRC1 and HLA-E-CD94:NKG2A interactions indicate that HLA-E overexpression on these myeloid cells directly engages the inhibitory receptors on NK_FCGR3A cells. This specific myeloid-NK cell crosstalk likely drives the functional exhaustion of NK cells, providing a mechanistic explanation for the reduced cytotoxic capacity observed in TNBC. Corroborating previous findings, the diminished expression of the NK cell activation receptor NKG2D (KLRK1) corresponds with reduced NK cell cytotoxicity in breast cancer [34]. This trend was also mirrored in our study, further highlighting the potential for an immunosuppressive tumor microenvironment within the TNBC group.

### Prognostic impact of NK_ISG15 cells or NK_FCGR3A cells in TNBC

**Fig 7:**
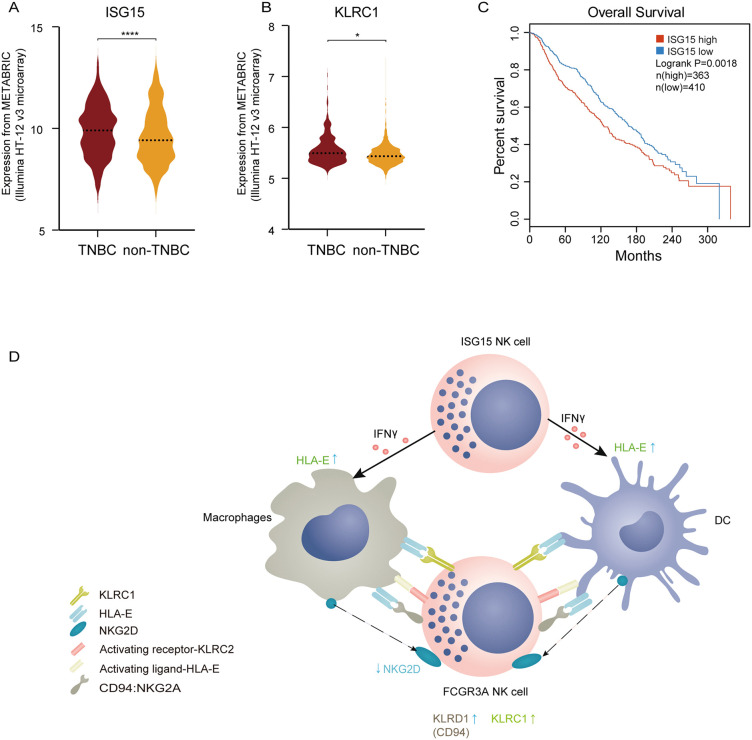
The potential significance of NK subtype gene signatures for breast cancer prognosis. (A/B) Expression of NK_ISG15 cell DEG (A: ISG15) and NK_FCGR3A cell DEG (B: KLRC1) by comparing TNBC and non-TNBC were analyzed in 2509 breast cancer patients. (dataset from METABRIC, Nature 2012 & Nat Commun 2016) (C) Prognostic value of ISG15 expression levels in tumor biopsies for 773 breast cancer patients (dataset from METABRIC, Nature 2012 & Nat Commun 2016). (D) Schematic illustration of the proposed mechanism of interactions between NK clusters and myeloid clusters in TNBC.

To further evaluate the clinical relevance of NK_ISG15- and NK_FCGR3A-associated molecular features in TNBC, we analyzed bulk transcriptomic expression profiles generated using Illumina microarray data from the METABRIC cohort, which included 320 TNBC patients and 2,189 non-TNBC patients [39]. Our aim was to quantify differences in the expression levels of DEGs within NK_ISG15 cells or NK_FCGR3A cells when comparing the TNBC to the non-TNBC cohort. Analysis revealed significantly higher expression of ISG15 and KLRC1 (NK_FCGR3A cells’ DEG) in the TNBC cohort relative to the non-TNBC cohort (Fig 7A-B). This finding implies that there is a dysregulated expression of genes critical to the cytotoxic function of NK_FCGR3A cells within the context of TNBC. This corroborates the single-cell data analysis results, bolstering the validity of the identified DEGs.

Survival analysis revealed that the expression level of ISG15 in tumor samples was significantly negatively correlated with patient survival in breast cancer (P = 0.0018) (Fig 7C). This finding underscores the substantial clinical prognostic value of ISG15. We performed survival analysis on NK cell marker genes (NCR1, NCR3, KLRB1, CD160, and PRF1) [14] (S7A Fig) and NK_FCGR3A cell marker genes (NCR1, NCR3, KLRB1, CD160, PRF1, GZMH, FGFBP2, SPON2, and NKG7) (S7B Fig). Our results demonstrated that high expression of NK_FCGR3A marker genes was associated with a better outcome (P = 0.0027) than that of NK cell marker genes (P = 0.021), indicating that the inclusion of these novel marker genes significantly enhances prognostic value. These findings highlight the critical importance of ISG15 gene expression and NK_FCGR3A cell infiltration levels as prognostic indicators, facilitating more precise predictions of patient outcomes in breast cancer, with particular relevance to differentiating TNBC from non-TNBC prognoses.

## Discussion

Natural killer (NK) cells are innate lymphocytes known for their potent cytotoxicity and have garnered increasing attention for their role in cancer immunity [40,41]. Investigating the biological complexity of human NK cells offers valuable insights for developing NK-focused cancer immunotherapy strategies [41]. Studies have demonstrated a correlation between NK cell activation and improved clinical outcomes in breast cancer patients. Furthermore, the integration of anti-NKG2A and anti-PD-L1 therapies has shown full responses in mouse models with heterogeneous MHC-I expression, underscoring their importance [15,42–44]. However, within the immunosuppressive TME, NK cells can become dysfunctional due to exposure to inhibitory molecules, contributing to immune escape [31,45,46]. Current biomarkers, including the presence of tumor-infiltrating immune cells, have shown limited utility in predicting immune checkpoint inhibitor (ICI) treatment outcomes. Consequently, it is vital to identify functionally impaired NK clusters and novel biomarkers indicative of TNBC progression to enhance the antitumor efficacy of NK-targeting strategies.

ScRNA-seq is an effective method to study the heterogeneity of NK cell clusters in breast cancer, particularly in terms of infiltration ratios and functions [8–10]. Previous studies have identified two major NK cell clusters and described their functions based on marker genes, but the mechanisms of transcription factor regulation, developmental maturation, and cellular interactions remain unclear. Comprehensive exploration of the functional significance of NK cells in breast cancer is incomplete, potentially due to low NK cell infiltration within tumors [17,27,30], the diversity of functional NK cell subtypes [29,31], and heterogeneity among breast cancer patients [47–49]. Identifying therapeutic targets for NK cells in breast cancer thus remains challenging. Moreover, current studies primarily focus on NK cells in overall breast cancer, whereas NK cells are more complex in TNBC tumors compared to non-TNBC tumors, necessitating further identification of NK clusters specifically in TNBC.

Our study leveraged scRNA-seq data from multiple breast cancer studies to perform an in-depth single-cell analysis of NK cell phenotypes and functions, comparing TNBC and non-TNBC tumors. We identified three distinct NK cell clusters: NK_XCL1, NK_FCGR3A, and NK_ISG15. The NK_FCGR3A cluster, characterized by high expression of cytotoxic markers such as GZMH, FGFBP2, and FCGR3A, indicates robust cytotoxic capabilities. NK_ISG15 cells, enriched in genes associated with type I interferon signaling (ISG15, IFI16, IFIT3, IFI6, and ISG20), suggest an involvement in innate immune responses. Crucially, our results reveal a significant reduction in the proportion of cytotoxic NK_FCGR3A cells in TNBC tissues. Pseudotime analysis further suggests an altered organization of NK-cell transcriptional states in TNBC, leading to an accumulation of partially immature NK cells, correlating with increased metastases and tumor growth [11]. The marked depletion of cytotoxic NK_FCGR3A cells in TNBC highlights a distinctive immunosuppressive feature of the TNBC microenvironment and may contribute to its poorer clinical outcomes.

The IFN-responsive NK_ISG15 state identified in our study may have functional implications beyond a simple inflammatory signature. Recent evidence indicates that ISG expression in NK cells is not exclusively driven by classical IFN-dependent induction, but can also be associated with NK-cell differentiation states and fate-determining transcriptional programs. In particular, Keller et al. showed that conserved ISG programs in NK cells were linked to NK-cell differentiation and were validated at both transcriptomic and protein levels, suggesting that ISG-high NK-cell states may reflect broader state organization rather than acute IFN stimulation alone [50]. This finding is relevant to our identification of NK_ISG15 cells in TNBC, as it supports a more nuanced interpretation of this subset as an ISG-high transcriptional state emerging in the inflammatory tumor microenvironment. In parallel, sustained type-I-IFN signaling in tumors has been reported to reshape intratumoral NK-cell programs and may be associated with reduced effector function in some contexts [51,52]. Therefore, NK_ISG15 cells may represent an ISG-high, IFN-responsive NK-cell state with potential functional impairment under chronic tumor-associated inflammatory pressure. Notably, the partial rescue of IFN-associated NK-cell dysfunction by IL-15 reported in previous studies [51,52] suggests that such states may retain a degree of functional plasticity, although this possibility was not directly tested in our study. Complementarily, ISG15 itself may also participate in tumor-promoting immune crosstalk. Extracellular or tumor-derived ISG15 has been reported to engage LFA-1 on macrophages and promote M2-like polarization, while analogous ISG15-related interactions in stromal cells may contribute to glycolytic reprogramming and a protumor niche [5,53,54]. Together with our survival analysis showing that higher ISG15 expression was associated with poorer prognosis, these observations suggest that NK_ISG15 may mark an inflammatory NK-cell state connected to tumor-associated immune suppression. However, because our findings are based on transcriptomic inference, the functional status and reversibility of NK_ISG15 cells should be considered hypothesis-generating and require further validation using protein-level, spatial, and functional assays.

As breast cancer progresses, there is a notable decrease in the expression of activating NK cell receptors such as NKG2D (KLRK1) and FCGR3A, coupled with an increase in inhibitory receptors like NKG2A (KLRC1), which correlates with diminished NK cell functionality, particularly cytotoxicity [17]. Elevated NKG2A expression is observed in advanced stages of breast cancer [55]. Previously scRNA sequencing data analysis of human TNBC revealed a high enrichment of NKG2A in NK cells [9]. In our study, we specifically observed an upregulation of the inhibitory receptor gene KLRC1 in NK_FCGR3A cells within the TNBC tumor, leading to an enrichment of negative immune regulatory response genes, thereby constraining their anti-tumor activity. KLRC1 may therefore serve as a relatively specific marker of TNBC-associated NK_FCGR3A state.

Previous studies have reported that KLRC1 knockout can overcome HLA-E-mediated inhibition and enhance NK cell antitumor activity against solid tumors [56,57]. Our cellular interaction analysis revealed a widespread upregulation of co-inhibitory checkpoints (KLRC1, CD94:NKG2A, HLA-E) in the TNBC group, which is linked to elevated interferon-related gene expression in NK_ISG15 cells. We hypothesize that this upregulation drives macrophages and DCs to overexpress HLA-E [58–60], fostering strong interactions between HLA-E and its receptors, KLRC1 and CD94:NKG2A, limiting the immune recognition capacity of NK cells in TNBC patients [57,61] (as proposed in Fig 7D). Otherwise, we demonstrate the prognostic impact of NK_ISG15 cells and NK_FCGR3A cells in breast cancer patients. This finding indicates that NKG2A-targeted therapy might have particularly high potential in TNBC.

The pivotal role of immune checkpoint inhibitors in anti-tumor therapy is well-established, and targeted inhibitors against NKG2A are being developed to provide new options for immunotherapy across various tumor types. Clinical trials involving Monalizumab (anti-NKG2A) have highlighted the efficacy of NKG2A-targeting inhibitors in tumor immunotherapy (NCT03822351 [62], NCT02643550 [63], NCT02671435 [64]), particularly when combined with other agents. Interest in NKG2A-targeting inhibitors in breast cancer treatment is exemplified by trials (NCT04307329) [65]. Trials approved by the National Medical Products Administration (NMPA) last year are investigating BioRay BRY805 and HyaMab HY-0102 in advanced solid tumors. Anti-NKG2A antibodies represent a promising immunotherapeutic strategy, offering hope for overcoming the challenges of current therapies in breast cancer. In addition to monoclonal antibody blockade, emerging modalities such as cold atmospheric plasma (CAP) are being explored for their potential to modulate the tumor microenvironment. Preliminary multi-omics data from the CAPmed-BC database suggest that CAP may influence molecular programs relevant to TNBC [66]. In our exploratory analysis of CAPmed-BC data, ISG15 transcript expression in breast cancer cell lines showed significant differences both before and after CAP treatment and between TNBC and non-TNBC models. Moreover, proteomic data derived from mouse cell line-derived xenograft (CDX) models indicated that NCAM1 was altered after CAP treatment in TNBC models. Nevertheless, these findings should be interpreted cautiously, as the available datasets were mainly derived from tumor cell lines and do not directly capture NK-cell states or NK–tumor microenvironment interactions. Therefore, the specific effects of CAP on the NKG2A–HLA-E axis, and its potential synergy with NKG2A-targeted blockade to restore NK-cell functionality, remain to be experimentally determined.

This study has limitations. First, although we integrated three public datasets, the final NK-focused cohort comprised only 25 untreated samples (15 TNBC and 10 non-TNBC), which may not fully capture the complete inter-patient heterogeneity of NK cells, particularly for rare subsets given the relatively low abundance of NK cells in the tumor microenvironment. Second, the exclusion of samples with <1% NK cells or fewer than 30 NK cells, while necessary to ensure the robustness of NK subtype analysis, may introduce selection bias by preferentially retaining tumors with relatively higher NK-cell infiltration and underrepresenting NK-poor or immune-excluded tumors. Third, our analysis is restricted to untreated primary tumor tissues and does not include treated or metastatic samples. The NK-cell states described here may not fully represent the phenotypic and functional heterogeneity of NK cells across different stages of disease progression or treatment contexts. Fourth, our analysis is based primarily on single-cell transcriptomic data, without matched proteomic or spatial validation. Single-cell transcriptomic data provide important insights into cell states and regulatory programs, but they do not directly measure NK-cell killing capacity, cytokine secretion, or protein-level checkpoint expression. Therefore, further validation using flow cytometry, cytotoxicity assays, single-cell proteomic approaches such as CITE-seq, and spatially resolved methods is necessary to strengthen these conclusions and refine the biological interpretation of these NK-cell states. In particular, the possibility that NK_ISG15 cells exhibit reduced cytotoxic activity or exhaustion-like features remains based on transcriptomic inference and requires further validation.

Despite these limitations, our study provides a comprehensive overview of the transcriptomic landscape of NK cells within the breast cancer TME, revealing significant heterogeneity between TNBC and non-TNBC. Integration of multiple high-quality datasets strengthened the robustness of the analysis, and the prognostic associations of ISG15 and NK_FCGR3A-related gene signatures highlight clinically relevant features of NK-cell dysfunction in breast cancer. Future studies in larger and more diverse cohorts, together with functional and spatial validation, will be necessary to refine the prognostic and therapeutic implications of these NK-cell states.

## Supporting information

S1 Fig Sample filtering and data integration. Related to Fig 1. (A) A schematic outline depicting the process of refining our dataset. (B) UMAP visualization of the unintegrated single-cell transcriptomes before batch correction, colored by sample origin. (C) UMAP to depict the tissue origins of the clusters, illustrating no obvious batch effect in this integrated atlas.
(TIF)

S2 Fig Identification of NK subcluster of integrated atlas of TME from breast cancer. Related to Fig 2. (A) UMAP projections showing 27 clusters from 130 breast cancer samples. (B) UMAP to depict NK subtype markers (GNLY and NKG7). (C) Violin plots of NK subtype markers (GNLY and NKG7) (columns) for clusters (rows) in 27 clusters.
(TIF)

S3 Fig Slingshot-inferred pseudotime trajectories of NK-cell subtypes in breast cancer. Related to Fig 2. (A-C) Slingshot pseudotime analysis showing the putative transcriptional-state transitions among NK_XCL1, NK_FCGR3A, and NK_ISG15 cells in breast cancer. (A) Slingshot-inferred trajectory of all NK cells from breast cancer samples. (B) Slingshot-inferred trajectory of NK cells from TNBC samples. (C) Slingshot-inferred trajectory of NK cells from non-TNBC samples. These analyses suggest potential state transitions among NK-cell subtypes, although pseudotime inference should not be interpreted as direct evidence of developmental lineage without experimental validation.
(TIF)

S4 Fig Heterogeneity in the molecular features of NK cells in TME of TNBC and non-TNBC. Related to Fig 4. GO (top) and KEGG (bottom) pathway annotation of gene signatures of NK cells in TNBC and non-TNBC.
(TIF)

S5 Fig Identification of T clusters and myeloid clusters of integrated atlas of TME from breast cancer. Related to Fig 5. (A/B) UMAP to depict the T clusters (A)/ myeloid (B) clusters from 25 breast cancer samples, illustrating no obvious batch effect in this integrated atlas. (C) Violin plots of markers (columns) for clusters (rows) in T clusters and myeloid clusters. Violin plots are colored by subtypes. (D/E) Boxplots of the cell proportions of T clusters (D), myeloid clusters (E) in TNBC and non-TNBC.
(TIF)

S6 Fig Cell-to-cell type interactions by cellPhoneDB in TNBC and non-TNBC. Related to Fig 5. (A/B) Heatmap showing the number of each cell-to-cell type interactions in TNBC (A) and non-TNBC (B).
(TIF)

S7 Fig The potential significance of NK_FCGR3A cell gene signature for breast cancer prognosis. Related to Fig 7. (A) Prognostic value of NK cell signature (NCR1, NCR3, KLRB1, CD160, PRF1) for overall survival of breast cancer patients (dataset from TCGA). (B) Prognostic value of NK_FCGR3A cell signature (NCR1, NCR3, KLRB1, CD160, PRF1, GZMH, FGFBP2, SPON2, NKG7) for overall survival of breast cancer patients (dataset from TCGA).
(TIF)

S1 Table Marker gene list of NK subtype in breast cancer. (XLSX)

S2 Table GO/KEGG analysis result of gene signatures in TNBC and non-TNBC. (A/B) GO/KEGG analysis result of gene signatures of NK cells in TNBC and non-TNBC. (C) GO analysis result of gene signatures of NK_XCL1 cells in TNBC and non-TNBC. (D/E) GO/KEGG analysis result of gene signatures of NK_FCGR3A cells in TNBC and non-TNBC. (F/G) GO/KEGG analysis result of gene signatures of NK_ISG15 cells in TNBC and non-TNBC.
(XLSX)
