# Supplementary figures and images for: Single-cell transcriptomic analysis reveals the immunosuppressive status of NK cell subpopulations in TNBC
Source: PLoS One. 2026 Jul 23;21(7):e0354524. doi: 10.1371/journal.pone.0354524 (PMC13395410; doi:10.1371/journal.pone.0354524)

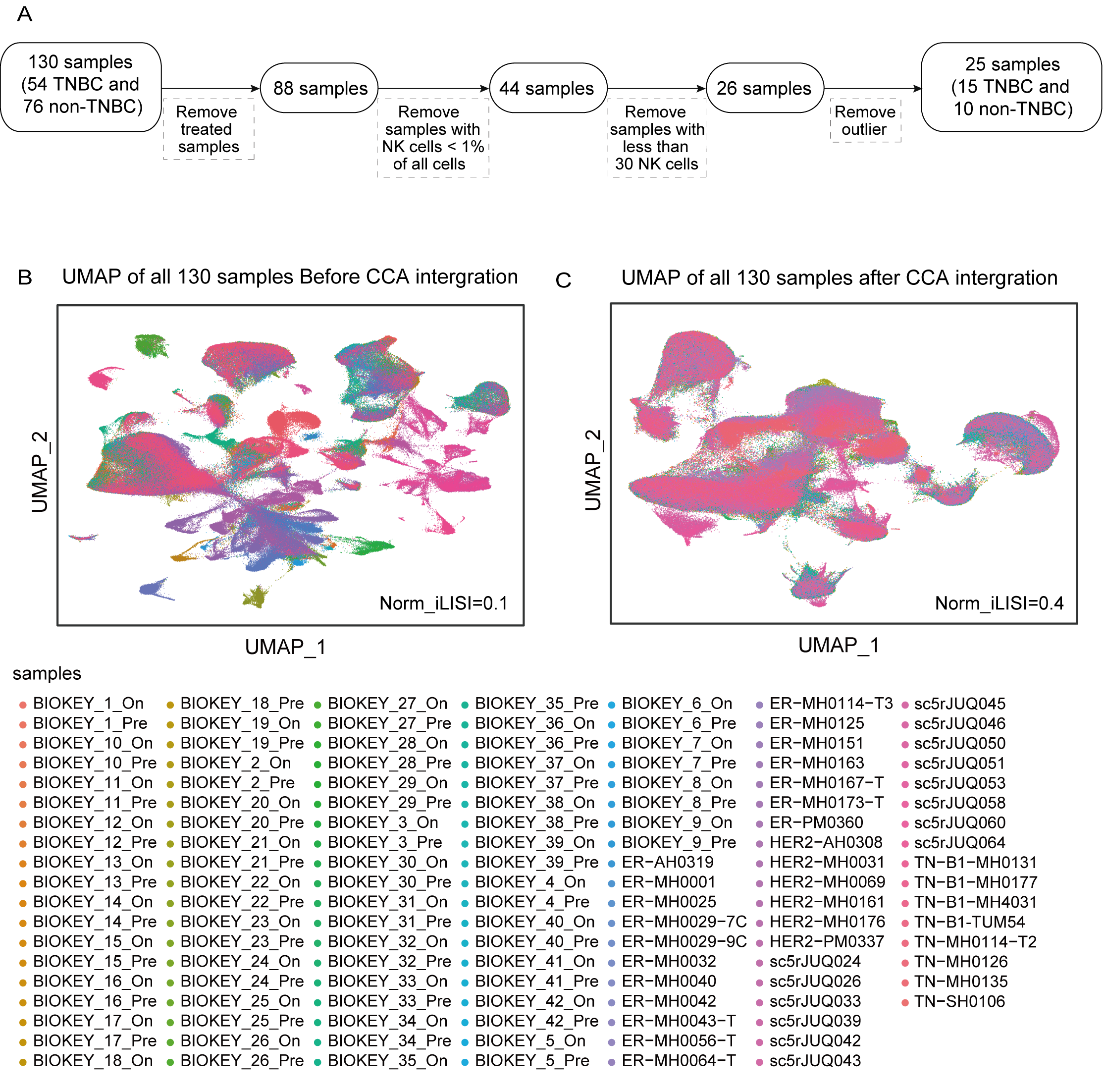

Supplement: S1 Fig — Related to Fig 1. (A) A schematic outline depicting the process of refining our dataset. (B) UMAP visualization of the unintegrated single-cell transcriptomes before batch correction, colored by sample origin. (C) UMAP to depict the tissue origins of the clusters, illustrating no obvious batch effect in this integrated atlas. (TIF) [file pone.0354524.s001.tif]

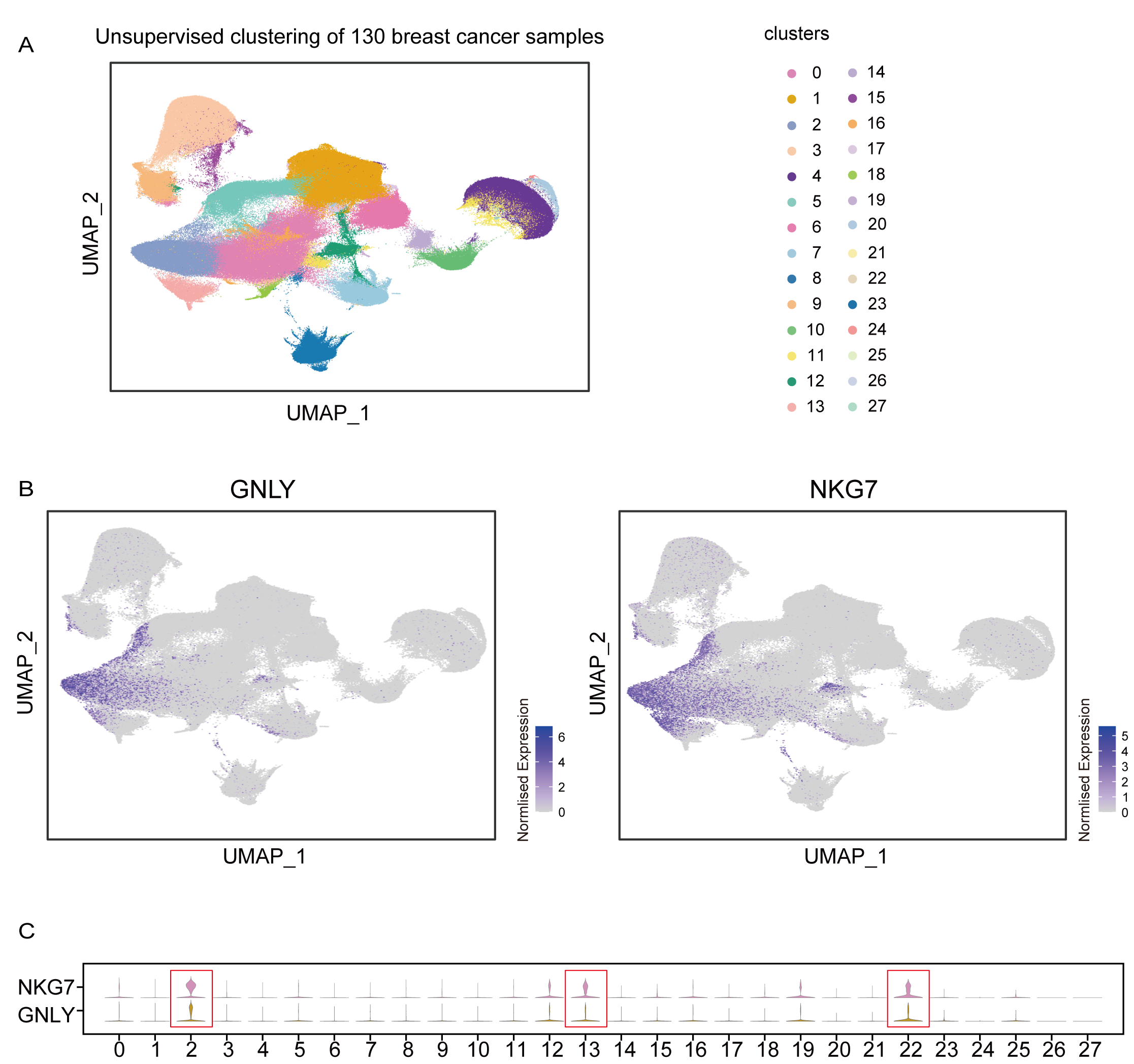

Supplement: S2 Fig — Related to Fig 2. (A) UMAP projections showing 27 clusters from 130 breast cancer samples. (B) UMAP to depict NK subtype markers (GNLY and NKG7). (C) Violin plots of NK subtype markers (GNLY and NKG7) (columns) for clusters (rows) in 27 clusters. (TIF) [file pone.0354524.s002.tif]

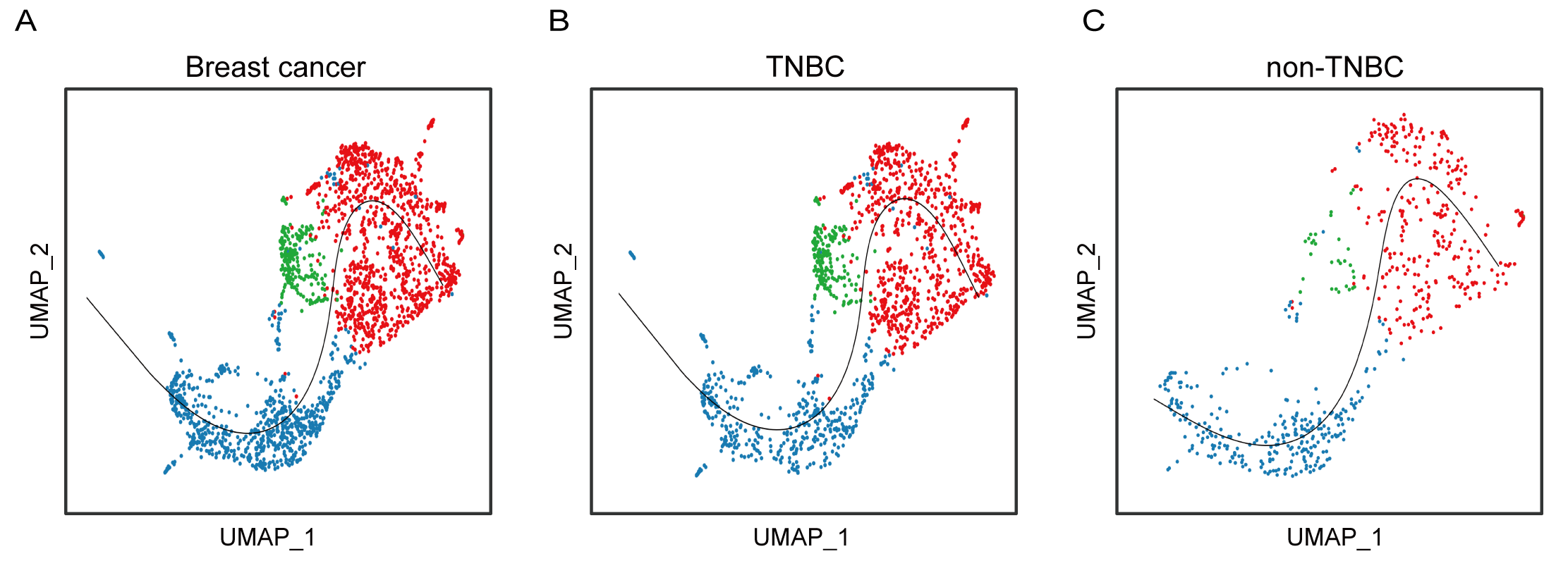

Supplement: S3 Fig — Related to Fig 2. (A-C) Slingshot pseudotime analysis showing the putative transcriptional-state transitions among NK_XCL1, NK_FCGR3A, and NK_ISG15 cells in breast cancer. (A) Slingshot-inferred trajectory of all NK cells from breast cancer samples. (B) Slingshot-inferred trajectory of NK cells from TNBC samples. (C) Slingshot-inferred trajectory of NK cells from non-TNBC samples. These analyses suggest potential state transitions among NK-cell subtypes, although pseudotime inference should not be interpreted as direct evidence of developmental lineage without experimental validation. (TIF) [file pone.0354524.s003.tif]

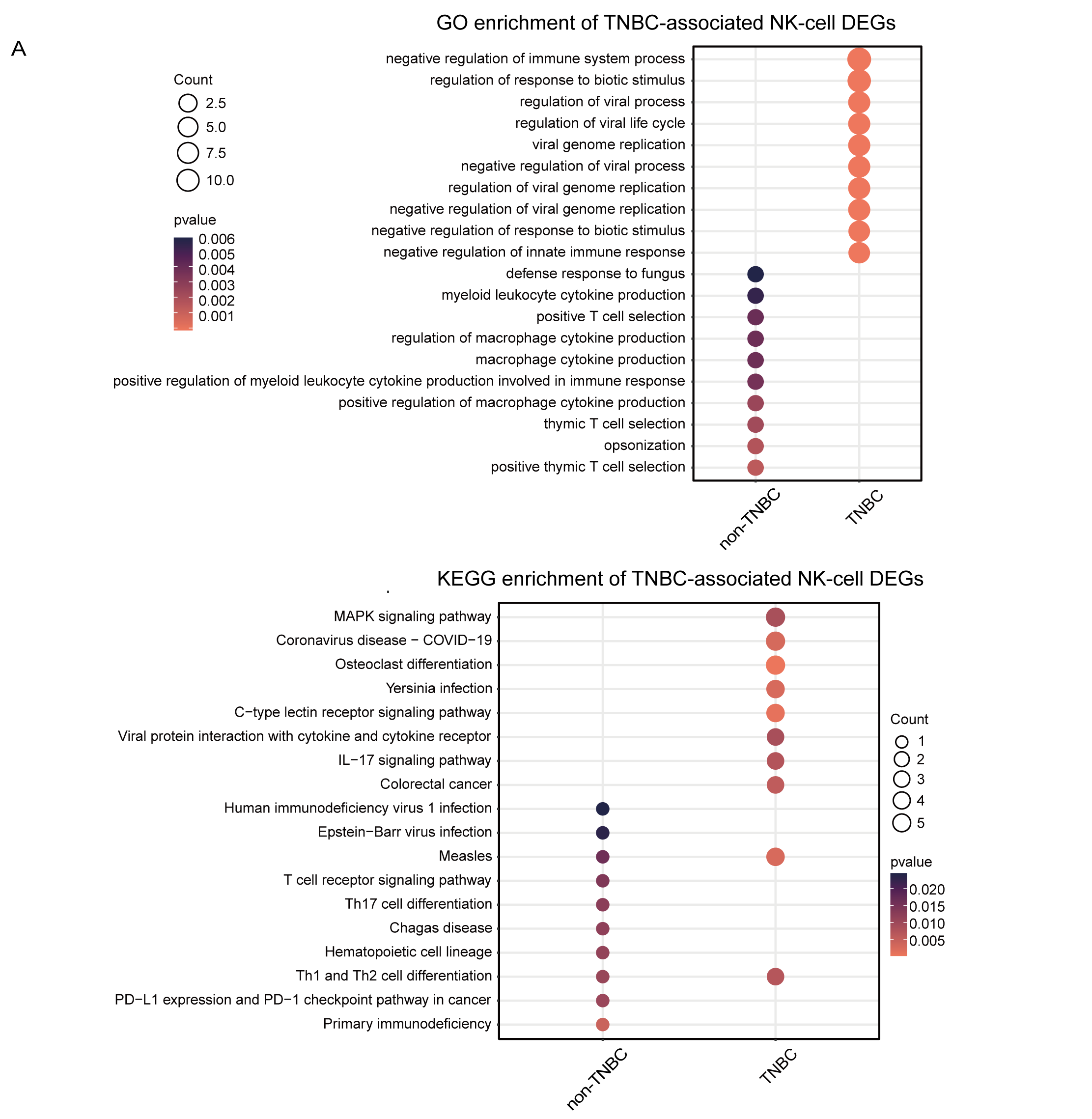

Supplement: S4 Fig — Related to Fig 4. GO (top) and KEGG (bottom) pathway annotation of gene signatures of NK cells in TNBC and non-TNBC. (TIF) [file pone.0354524.s004.tif]

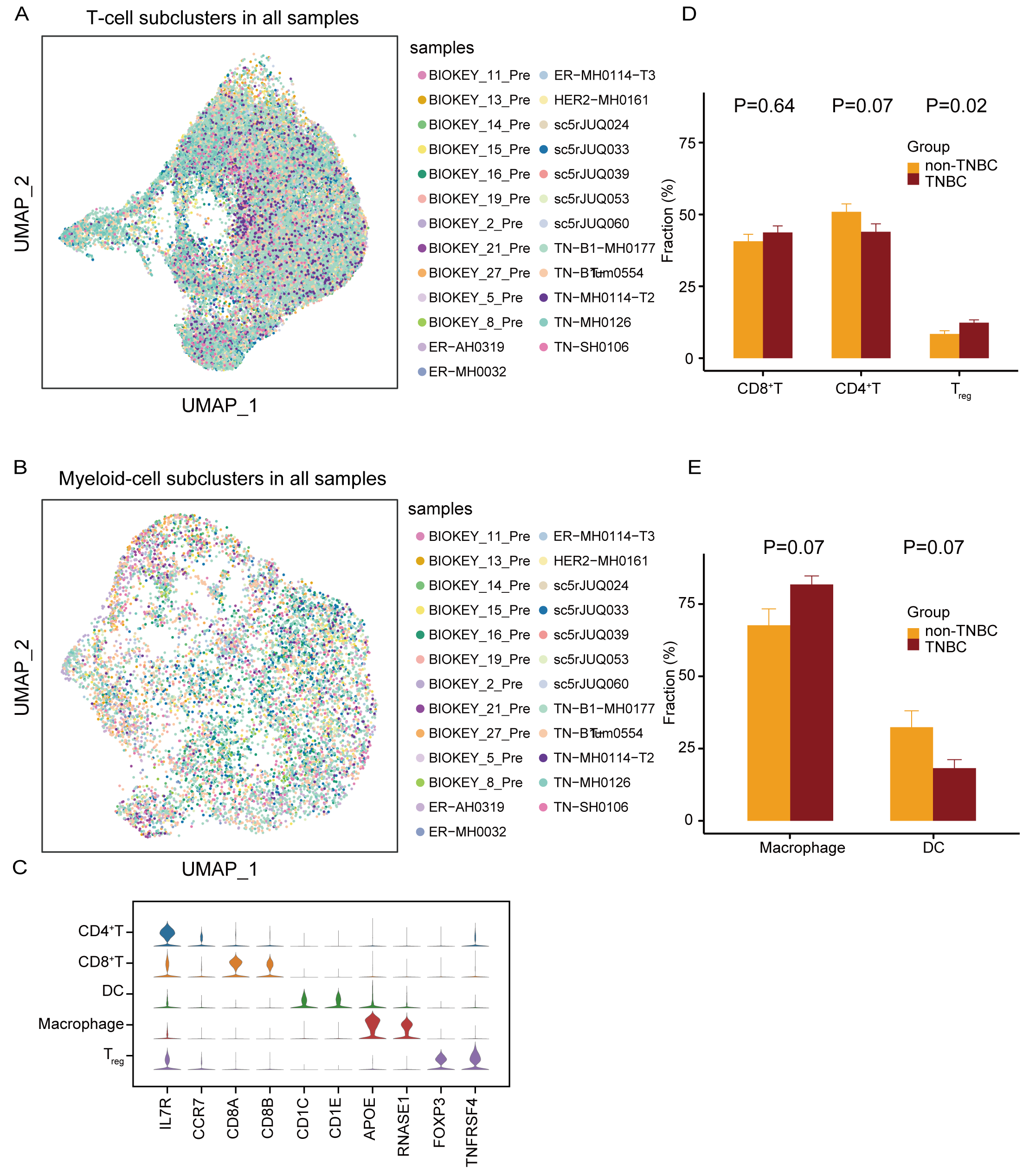

Supplement: S5 Fig — Related to Fig 5. (A/B) UMAP to depict the T clusters (A)/ myeloid (B) clusters from 25 breast cancer samples, illustrating no obvious batch effect in this integrated atlas. (C) Violin plots of markers (columns) for clusters (rows) in T clusters and myeloid clusters. Violin plots are colored by subtypes. (D/E) Boxplots of the cell proportions of T clusters (D), myeloid clusters (E) in TNBC and non-TNBC. (TIF) [file pone.0354524.s005.tif]

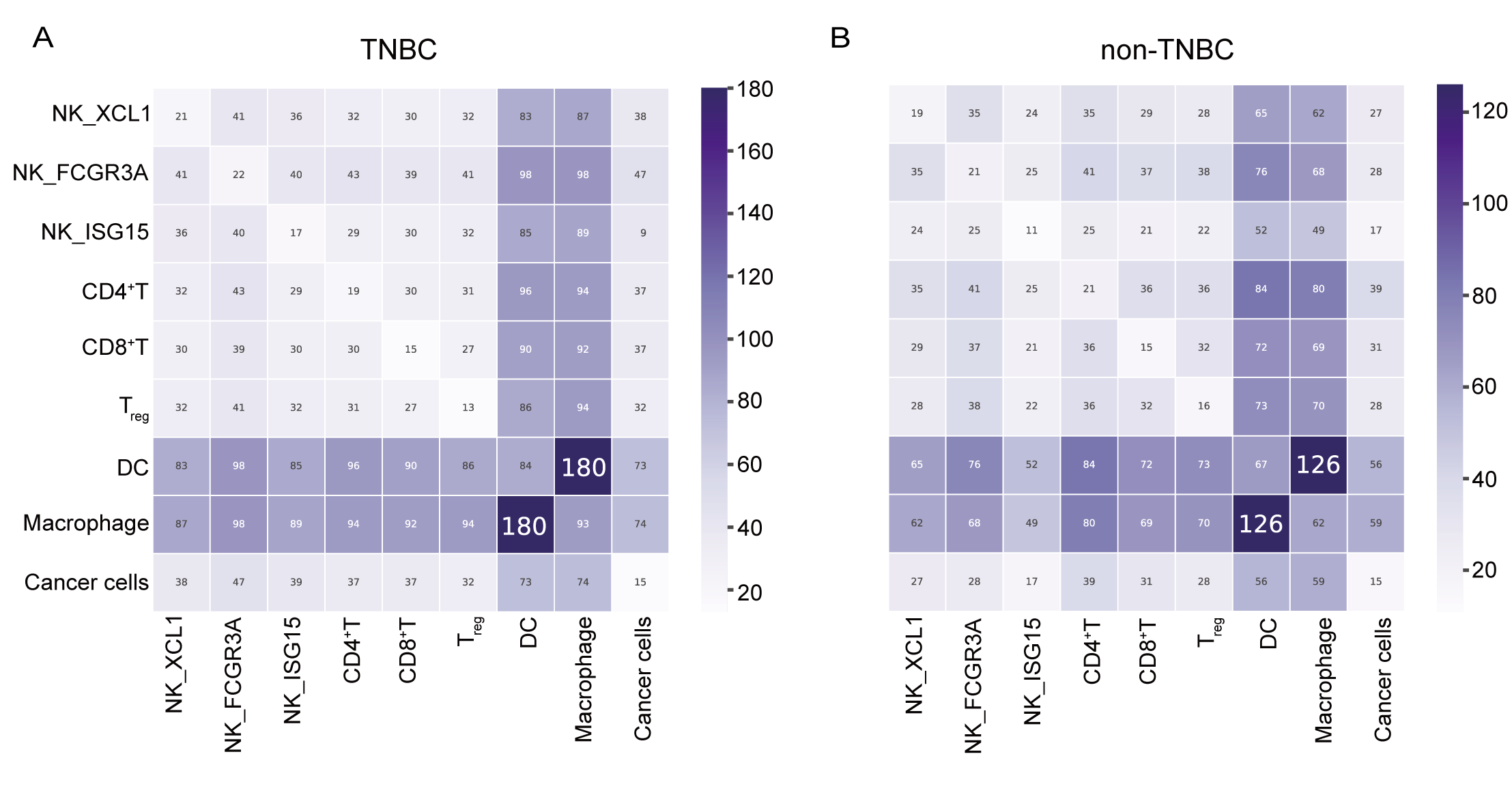

Supplement: S6 Fig — Related to Fig 5. (A/B) Heatmap showing the number of each cell-to-cell type interactions in TNBC (A) and non-TNBC (B). (TIF) [file pone.0354524.s006.tif]

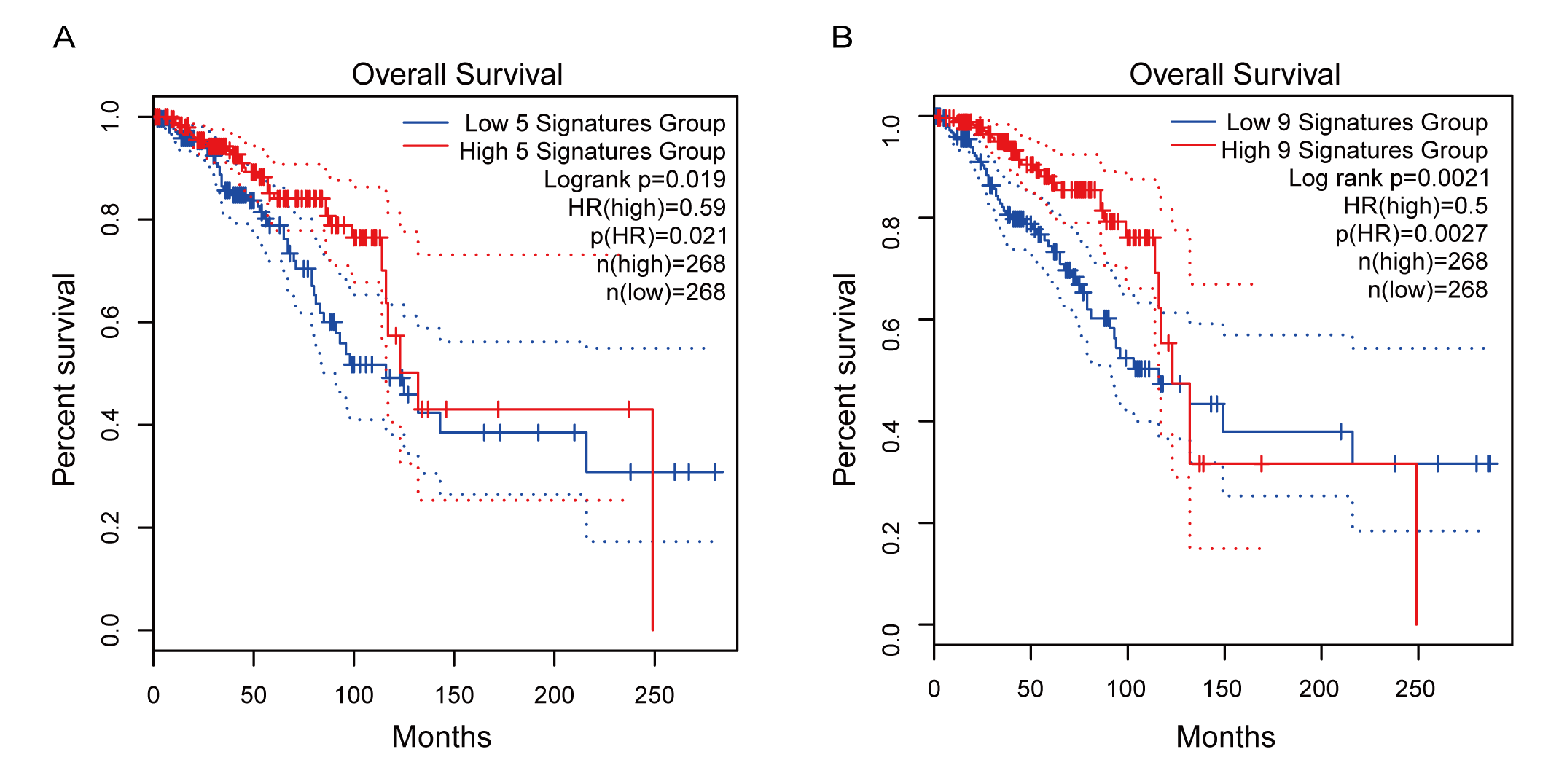

Supplement: S7 Fig — Related to Fig 7. (A) Prognostic value of NK cell signature (NCR1, NCR3, KLRB1, CD160, PRF1) for overall survival of breast cancer patients (dataset from TCGA). (B) Prognostic value of NK_FCGR3A cell signature (NCR1, NCR3, KLRB1, CD160, PRF1, GZMH, FGFBP2, SPON2, NKG7) for overall survival of breast cancer patients (dataset from TCGA). (TIF) [file pone.0354524.s007.tif]
